# Presidential address 2026: celebrating academic excellence and expanding computer-based testing across health professions

**DOI:** 10.3352/jeehp.2026.23.1

**Published:** 2026-01-09

**Authors:** Hyunjoo Pai

**Affiliations:** President, Korea Health Personnel Licensing Examination Institute, Seoul, Korea; The Catholic University of Korea, Korea

Dear *Journal of Educational Evaluation for Health Professions* community,

As we step into 2026, I am filled with deep gratitude for all that we have accomplished together, as well as genuine excitement for the opportunities that lie ahead.

## A year of remarkable achievements

Our collective efforts have elevated the *Journal of Educational Evaluation for Health Professions* (JEEHP) to new levels of academic excellence. The numbers tell a compelling story: our 2024 CiteScore of 16.4 places us in the 99th percentile among 1,620 education journals worldwide and in the 97th percentile among general medicine journals. In addition, our SCImago Journal Rank of 2.068 positions us fourth among 348 Korean journals. Yet beyond these quantitative indicators lies something even more meaningful: the real-world impact of the research published within these pages.

## Power of community

These achievements belong to you. They reflect the contributions of our authors, who pose innovative research questions and pursue rigorous scholarship; our reviewers, who strengthen every manuscript through their expertise and constructive feedback; our editors and editorial team, who uphold the highest standards while cultivating an environment in which new ideas can flourish; and our global readership, whose engagement and citations extend the reach and relevance of published work far beyond our initial expectations.

## Innovation in health personnel evaluation

**Fig. 1.**
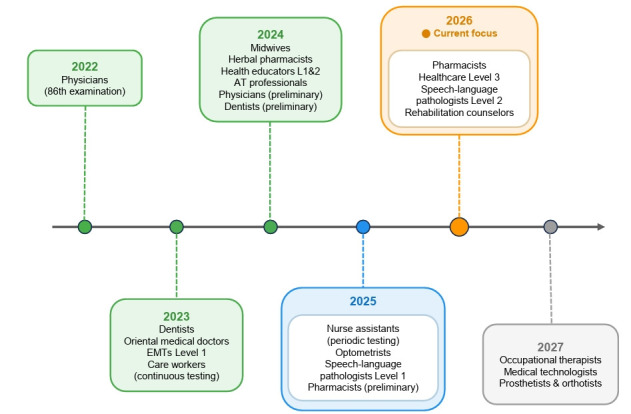
AT, assistive technology; EMTs, emergency medical technicians.

The Korea Health Personnel Licensing Examination Institute continues to advance the future of professional assessment. Building on the successful implementation of computer-based testing (CBT) for the Korean Medical Licensing Examination in 2022 [1], many other licensing examinations have since been administered using CBT. These include the licensing examinations for dentists, midwives, oriental medical doctors, emergency medical technicians, health educators, herbal pharmacists, care workers, assistive technology professionals, nurse assistants, optometrists, and speech-language pathologists [2,3]. We are further expanding CBT in 2026 to include pharmacists, health educators, speech-language pathologists, and rehabilitation counselors (Fig. 1, Supplement 1). This evolution reflects our shared commitment to excellence in health professions education and evaluation.

## Looking ahead

JEEHP’s mission remains clear: to serve as a trusted platform for groundbreaking research in health education and evaluation. We remain firmly committed to openness, academic rigor, and global accessibility. Every submission, review, and citation strengthens the bridge between research and practice, reinforcing the journal’s role in advancing evidence-informed education.

In 2026, we will continue to expand our international reach and impact. We aim to support emerging voices alongside those of established scholars, facilitate conversations that advance health professions education worldwide, and ensure that authors’ research receives the visibility and recognition it rightfully deserves.

## An invitation

Your research matters. Your insights drive progress in health education. We invite you to continue sharing your work, engaging with fellow scholars, and contributing to the vibrant intellectual community that defines JEEHP.

Thank you for your partnership, trust, and dedication to advancing our field. Here is to a year filled with discovery, innovation, and meaningful impact.

Wishing you good health, inspiration, and academic success in 2026.

With gratitude and anticipation.

## Supplementary materials

Supplement 1. Year of implementation (established and scheduled) of computer-based testing for health personnel licensing examinations in Korea.
