# Supplementary material for: Presidential address 2026: celebrating academic excellence and expanding computer-based testing across health professions
Source: J Educ Eval Health Prof. 2026 Jan 9;23:1. doi: 10.3352/jeehp.2026.23.1 (PMC12976626; doi:10.3352/jeehp.2026.23.1)
Supplement: Supplementary file 1 — Supplement 1. Year of implementation (established and scheduled) of computer-based testing for health personnel licensing examinations in Korea. [file jeehp-23-01-suppl1.docx]

**Supplement 1.** Year of implementation (established and scheduled) of computer-based testing for health personnel licensing examinations in Korea

| Category of health profession | Year of implementation | Examination session from | Testing format |
| --- | --- | --- | --- |
| Physicians | 2022 | The 86th national examination |  |
| Dentists | 2023 | The 75th national examination |  |
| Oriental medical doctors | 2023 | The 78th national examination |  |
| Emergency medical technician–paramedic | 2023 | The 29th national examination |  |
| Midwives | 2024 | The 35th national examination |  |
| Herbal pharmacists | 2024 | The 25th national examination |  |
| Health educators Level 1 & 2 | 2024 | The 15th national examination |  |
| Assistive technology professionals | 2024 | The 6th national examination |  |
| Physicians (preliminary) | 2024 | The 20th national examination |  |
| Dentists (preliminary) | 2024 | The 20th national examination |  |
| Care workers | 2023 | The 2023 qualification examination | Continuous testing |
| Nurse assistants | 2025 | The first half 2025 national examination | Periodic testing |
| Optometrists | 2025 | The 38th national examination |  |
| Speech-language pathologists Level 1 | 2025 | The 14th national examination |  |
| Pharmacists | 2026 | The 77th national examination |  |
| Health educators Level 3 | 2026 | The 17th national examination |  |
| Speech-language pathologists Level 2 | 2026 | The 15th national examination |  |
| Rehabilitation counselors Level 1 & 2 | 2026 | The 10th national examination |  |
| Pharmacists (preliminary) | 2026 | The 7th preliminary examination |  |
| Occupational therapists | 2027 | The 55th national examination |  |
| Medical technologists | 2027 | The 55th national examination |  |
| Prosthetist and orthotists | 2027 | The 28th national examination |  |
